# Plasmonic Heating-Promoted Photothermal Synthesis of α-Cyanoacrylonitriles Over Au/h-BN Catalysts

**DOI:** 10.3389/fchem.2021.732162

**Published:** 2021-09-08

**Authors:** Ce Liang, Yuanyuan Zhang, Bin Zhang, Xin-Miao Liu, Guo-Lin Gao, Jingyan Cao, Ping Xu

**Affiliations:** ^1^ MIIT Key Laboratory of Critical Materials Technology for New Energy Conversion and Storage, School of Chemistry and Chemical Engineering, Harbin Institute of Technology, Harbin, China; ^2^ Department of Medical Oncology, Harbin Medical University Cancer Hospital, Harbin, China

**Keywords:** plasmonic heating, photothermal catalysis, Au/h-BN, α-cyanoacrylonitriles, reaction mechanism

## Abstract

Plasmonic nanoparticle-involved materials play an essential role in the field of photothermal conversion. Herein, we report the application of photothermal heterogeneous catalysts consisting of gold nanoparticles decorated on defect-rich h-BN sheets (Au/h-BN) for the photocatalytic synthesis of α-cyanoacrylonitriles under mild conditions. It has been demonstrated the–NH_2_ groups present in the defect-rich h-BN act as the catalytically active sites, while plasmonic heating from the gold nanoparticles can drive the reaction by providing local heat. Au/h-BN catalyst can work for a broad substrate scope in the synthesis of α-cyanoacrylonitriles, and a plausible –NH_2_ group-involved reaction mechanism has been proposed. This work may open up new avenues in photothermal catalysis by combining plasmonic materials and catalytic sites in one system.

## Introduction

Thermal energy is one of the most commonly used energy sources in human life and production (Fan, 2017). Among various methods of obtaining thermal energy, photothermal conversion has a bright future due to its convenient, efficient, and green nature (Zhu et al., 2018). Photothermal conversion is usually accomplished by photothermal agents, which convert light energy into heat via light absorption and non-radiation processes. Particularly, plasmonic photothermal materials that rely on localized surface plasmon resonance (LSPR) exhibit remarkable advantages since the LSPR property can be easily manipulated by tuning their shape, size, composition and surrounding medium (Jauffred et al., 2019). For this reason, plasmonic photothermal materials have been broadly utilized in the areas of water recycling (Zhang et al., 2019), catalysis (Mateo et al., 2021), photothermal therapy (Cheng et al., 2014) etc. (Kim et al., 2019). Generally, LSPR generates heat to go through three processes: 1) The incident light resonates with the electron cloud of nanomaterials to generate a locally enhanced electromagnetic field (Kazuma and Kim, 2019). 2) The oscillation of free electrons rapidly decays through the formation of hot charge carriers (hot electrons and holes) (Zhang et al., 2018; Li and Jin, 2020). Notably, these hot carries can induce several reactions, such as ethylene epoxidation (Christopher et al., 2011), hydrogenation of carbonyl compounds (Landry et al., 2017), etc. (Dai et al., 2015; Yin et al., 2020). Our group also reported a number of studies on the dimerization reactions of 4-aminothiophenol or 4-nitrothiophenol to 4,4′-dimercaptoazobenzene (Kang et al., 2013; Xu et al., 2013; Kang et al., 2015; Shen et al., 2018; Liang et al., 2020; Li et al., 2021). 3) The photoexcited hot carriers evolve to a Fermi−Dirac distribution via electron−electron scattering (Jiang et al., 2018). Then heat is generated through electron−phonon scattering and eventually releases to the surrounding medium (Jauffred et al., 2019). In fact, plasmonic heating has been successfully utilized in some chemical transformations but relatively rare (Lemieux et al., 2006; Adleman et al., 2009; Zhao et al., 2015). For example, Boyd and co-workers reported the use of plasmonic heat generated around Au nanoparticles to promote steam reforming of ethanol to form CO_2_, CO and H_2_ (Adleman et al., 2009). Also, the group of Branda disclosed a thermally enhanced plasmonic photocatalysis of a retro Diels-Alder reaction using Au nanoparticles (Lemieux et al., 2006). Moreover, Xiong et al. described Pd-Ag alloy nanocages for the photothermally catalyzed hydrogenation of styrene (Zhao et al., 2015).

Encouraged by these findings, herein, we demonstrate gold nanoparticles on defect-rich h-BN sheets (Au/h-BN) as a photothermal catalyst for the synthesis of α-cyanoacrylonitriles. α-Cyanoacrylonitriles have played critical roles as synthetic intermediates (Cutrí et al., 1998), riot control agents (Jones, 1972), pre-polymers (Lee et al., 2007; Kharas et al., 2009), piezoelectric materials (Lee et al., 2003), and optoelectronic devices (Aloui et al., 2016). Moreover, in drug discovery, α-cyanoacrylonitriles are potential compounds with cytostatic (Latif et al., 1970), anti-inflammatory (Girgis et al., 2007), hypotensive (El-Sadek et al., 2007), and bronchodilatory (Girgis et al., 2015) properties. Therefore, studying the synthesis of α-cyanoacrylonitriles is of great importance for future applications. In terms of Au/h-BN, the Au nanoparticles act as plasmonic nanoheaters, which generate heat upon light irradiation and then transfer the heat to the defect-rich h-BN support containing active catalytic sites. This process can drive the cyanation reaction under mild conditions, which we believe can be applied in other photothermally driven organic syntheses.

## Experimental Section

### Preparation of Au Nanoparticles

The Au nanoparticles were synthesized according to a reported protocol (Frens, 1973). 100 ml of HAuCl_4_ solution (10^−2^ wt% in water) was added into a 250 ml three-necked round-bottomed flask and then heated with a heating mantle for 15 min under vigorous stirring. A condenser was utilized to prevent the evaporation of the solvent. After boiling (100°C) had commenced, 0.32 ml of sodium citrate (1 wt% in water) was injected. The color of the solution changed from yellow to bluish-gray and then to purplish-red in 30 min, indicating the formation of gold nanoparticles.

### Preparation of Defect-Rich h-BN

The defect-rich h-BN sheets were prepared as reported with some modifications (Feng et al., 2019). 5 g urea and 1 g boric acid were dissolved in 10 ml deionized water to form a homogeneous solution, which was heated to 80°C for recrystallization. A white crystalline powder was obtained upon evaporation of the solvent, which was then put into a horizontal tube furnace. After removal the air with nitrogen, the furnace was heated up to 800°C at a heating rate of 5°C/min and the powder was annealed at 800°C for 2 h under the protection of nitrogen. After that, pyrolysis product was obtained and washed with hot water to remove boron oxide and obtain defect-rich h-BN.

### Preparation of Au/h-BN Nanocatalysts

50 mg defect-rich h-BN was added into a 50 ml Au dispersion (0.1 mg/ml in water) and stirred for 24 h at room temperature. The resulting dispersed solution was filtered with a 0.1 μm pore size membrane to separate the unattached Au nanoparticles from the Au/h-BN nanocomposite.

### Characterization

X-ray diffraction (XRD) patterns were collected on a Rigaku D/MAXRC X-ray diffractometer (45.0 kV, 50.0 mA) ray diffractometer with Cu Kα radiation (λ = 0.15406 nm). Transmission electron microscopic (TEM) images were taken by an FEI Tecnai F20 operating at an accelerating voltage of 200 kV. The Fourier transform infrared (FT-IR) spectra were obtained on a Thermo Scientific Nicolet iS5 FT-IR spectrometer. X-ray photoelectron spectra (XPS) were recorded on a PHI-5700 ESCA system using Al Kα radiation as a source (hυ = 1,486.6 eV) and binding energy values were reported relative to the C 1s of surface adsorbed carbon (= 284.5 eV). UV-visible absorption spectra were analyzed were gained on a Persee TU-1901 spectrophotometer. Photothermal images were measured by a Fotric 225s infrared camera while illuminating the sample (2 mg h-BN and 2 mg Au/h-BN dispersed in 1 ml acetonitrile, respectively) with a 300 W Xe lamp (PLS-SXE300/300UV, wavelength: 330–2,500 nm, beam diameter: 30 mm). Flash column chromatography was performed using 200–300 mesh silica gel. All materials and solvents were used as received from commercial sources without further purification unless otherwise noted. ^1^H and ^13^C NMR spectra were obtained on a Bruker AV-400 or AV-600 instrument in CDCl_3_ or DMSO-d_6_ with TMS (SiMe_4_) as an internal standard, and chemical shift values were reported in ppm relative to dimethyl TMS (δ = 0.00 ppm) or DMSO (δ = 2.50 ppm) for ^1^H NMR, chloroform (δ = 77.0 ppm), or DMSO (δ = 39.5 ppm) for ^13^C NMR. The reported chemical shifts (δ) of ^13^C NMR were ^13^C{1H} proton-decoupled carbons data. The following abbreviations (or combinations thereof) were used to explain multiplicities: s = singlet, d = doublet, t = triplet, q = quartet, and m = multiplet.

### Computational Methods

All geometric optimization and energy analysis were performed utilizing the Gaussian09 software package. The DFT calculations were conducted using the B3LYP exchange-correlation functional. The 6-31G(d, p) basis set was chosen for C, H, O, B and N atoms. The model of the catalyst was simulated with 19 B atoms, 20 N atoms and 17 H atoms, where B and N atoms were alternately connected along with an amino group hanging on the edge. Adding hydrogen atoms at the end can avoid the unsaturated boundary effect. To facilitate the calculations of free energy change in the reactions, Au nanoparticles were omitted during the calculations because the experimental results showed that the Au nanoparticles only act as nanoheaters instead of catalytic sites.

### Photothermal Reaction Conditions

In a 10 ml vial, aldehydes (0.10 mmol), malononitrile (0.13 mmol), Au/h-BN (2.0 mg), and anhydrous MeCN (1.0 ml) were added in sequence under magnetic stirring, and then the vial was sealed. The system was evacuated by five freeze pump-thaw cycles and back-filled with N_2_. Then, the vial was irradiated by a Xe lamp for 18 h. After reaction completion, the vial contents were evaporated under reduced pressure. The residue was purified by precipitation thin-layer chromatography (PTLC) using PE/EtOAc (60:1 to 1:1 depending on the substrates) as the eluent to afford the desired product.

## Results and Discussion

### Synthesis and Characterization of Au/h-BN

**FIGURE 1:**
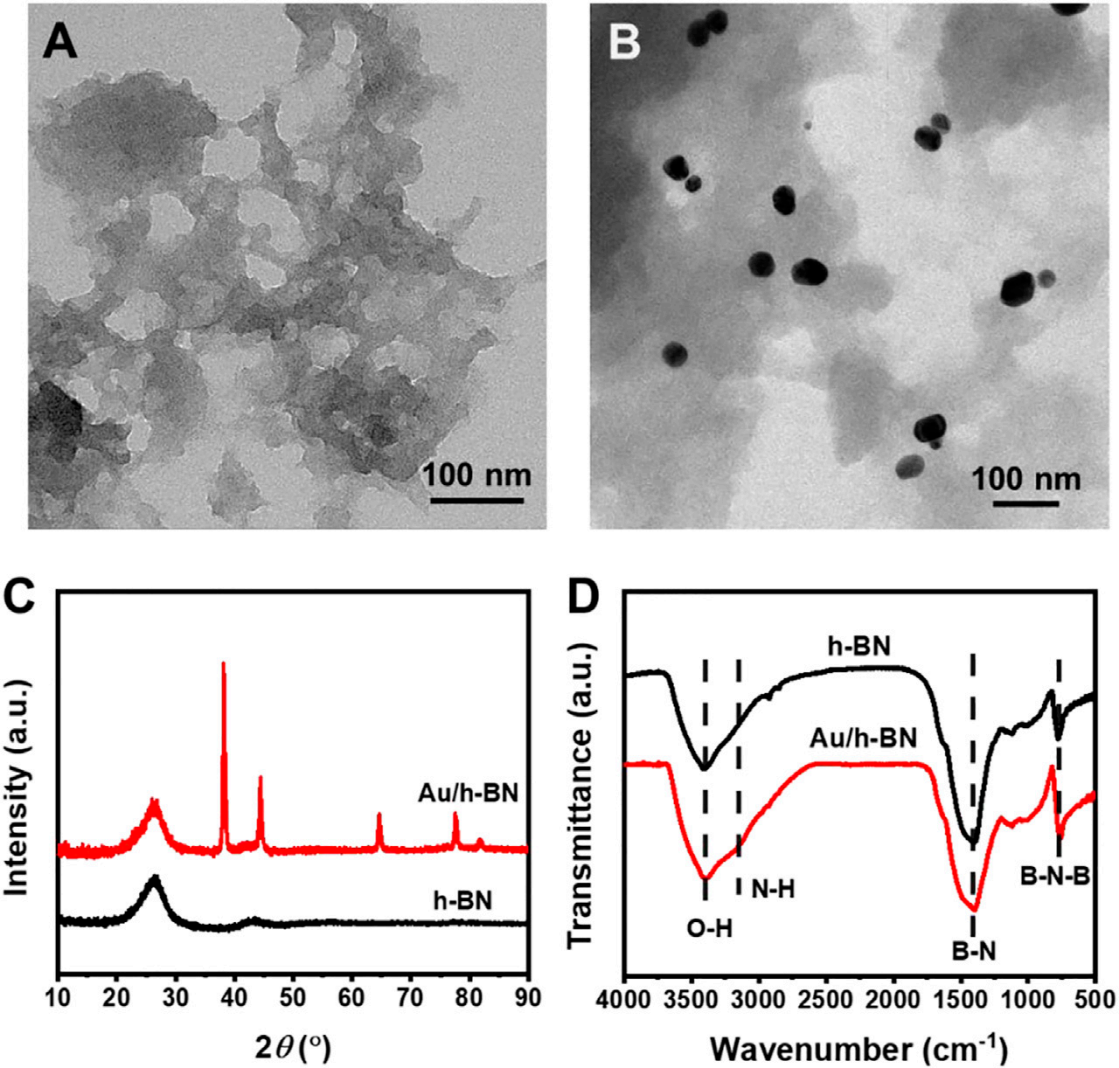
TEM images for **(A)** bare h-BN and **(B)** Au/h-BN composite. **(C)** XRD patterns and **(D)** FTIR spectra for Au/h-BN composite and bare h-BN.

Au/h-BN nanocatalysts were produced through a wet-impregnation strategy (see detail in Experimental Section). In brief, Au nanoparticles and defect-rich h-BN were first prepared separately and then combined to form the Au/h-BN composites. TEM was carried out to reveal the transparent nanosheet morphology of the synthesized h-BN, as shown in Figure 1A. Additionally, many pores are observed, which can serve as nanoreactors and thus are beneficial for heterogeneous catalysis. This porous structure implies that the synthesized h-BN is defect-rich because an ideal perfect h-BN is a flawless two-dimensional matrix. Furthermore, Supplementary Figure S1 in supporting information showed the morphology of Au nanoparticles was nanospheres with an average diameter of ∼30 nm. Notably, Figure 1B reveals that the h-BN surface is successfully decorated with Au nanoparticles, indicating the formation of Au/h-BN composite as expected. These results were supported by powder XRD patterns (Figure 1C). Two broad peaks at 26.6° and 43.5° can be indexed to h-BN (PDF#85-1068). The broad feature suggests its low crystallinity. Furthermore, the peaks at 38.2°, 44.4°, 64.6°, 77.5° and 81.7° in the Au/h-BN composite are from gold (PDF#89-3697). Fourier-transform infrared spectroscopy was carried out to investigate the chemical structures of samples (Figure 1D). The absorption peaks at 1,390 and 778 cm^−1^ are attributed to the in-plane B-N stretching vibration and the out-of-plane B-N-B bending bands, respectively (Weng et al., 2015). In addition, the bands at 3,180 and 3,400 cm^−1^ indicate the presence of amino and hydroxyl functional groups (Ou et al., 2017). This confirms that B and N atoms are not entirely cross-linked to form a perfect two-dimensional structure. In other words, the amino and hydroxyl groups are present as dangling bonds at the edge or pores of the defect-rich h-BN.

**FIGURE 2:**
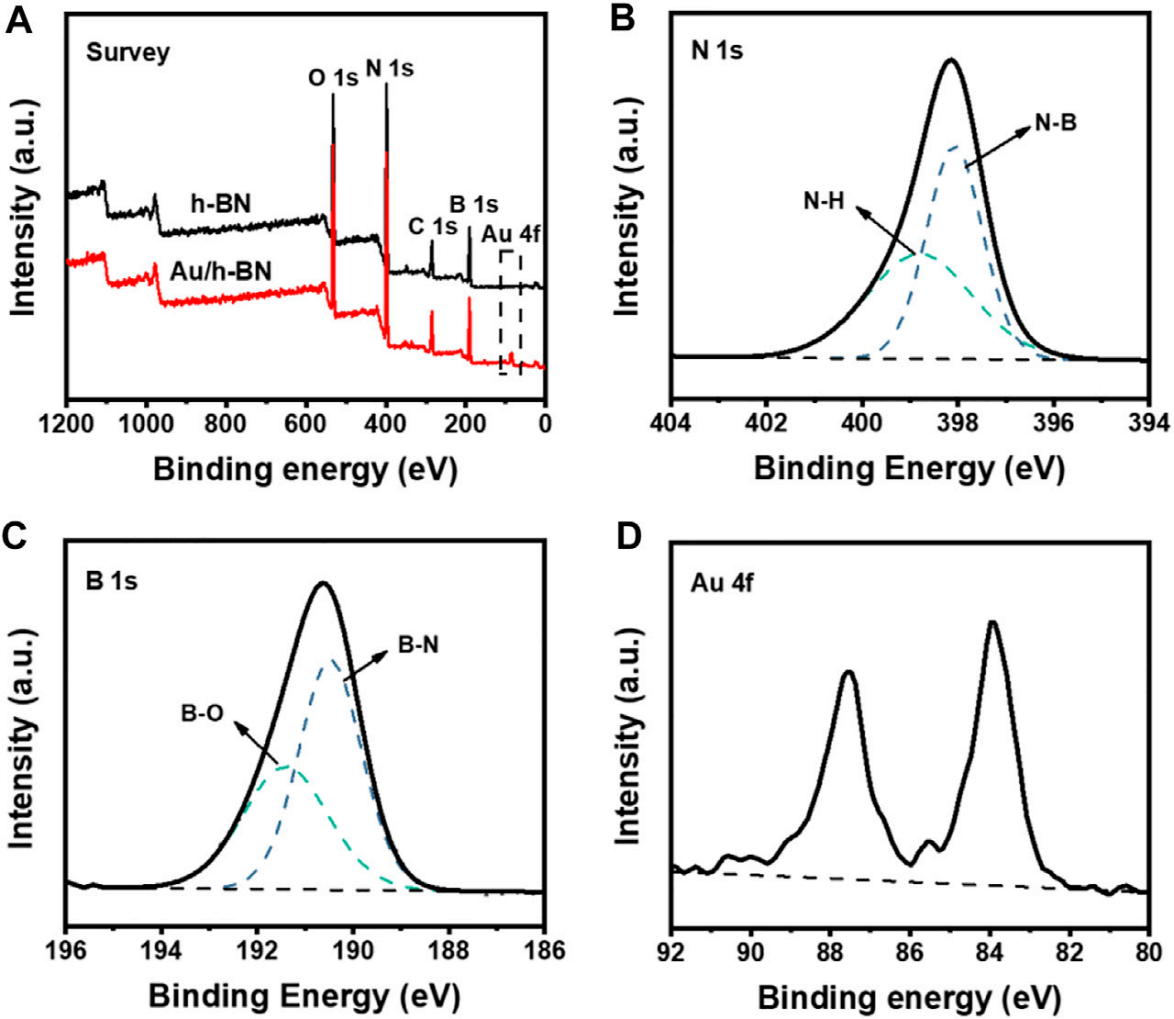
**(A)** XPS survey spectra for Au/h-BN composite and bare h-BN. **(B)** N 1s **(C)** B 1s and **(D)** Au 4f spectra for Au/h-BN composite.

The XPS spectra were also collected to study the bonding state of the Au/h-BN composite (Figures 2A–D). In the N 1s spectrum (Figure 2B), the main component centered at 398.1 eV corresponded to the N-B band, whereas the higher binding energy at 398.8 eV was caused by amino groups (Zhi et al., 2009). The B 1s spectrum was depicted in Figure 2C, and the dominant peak at 190.5 eV accounts for B-N bonds, and the shoulder peak at 191.4 eV is due to B-O bonds (Zhi et al., 2009; Perez et al., 2014; Liu et al., 2017). Besides the peaks of B and N, the signal of Au could be clearly observed from the Au/h-BN composite as compared to the bare h-BN (Figures 2A,D).

**FIGURE 3:**
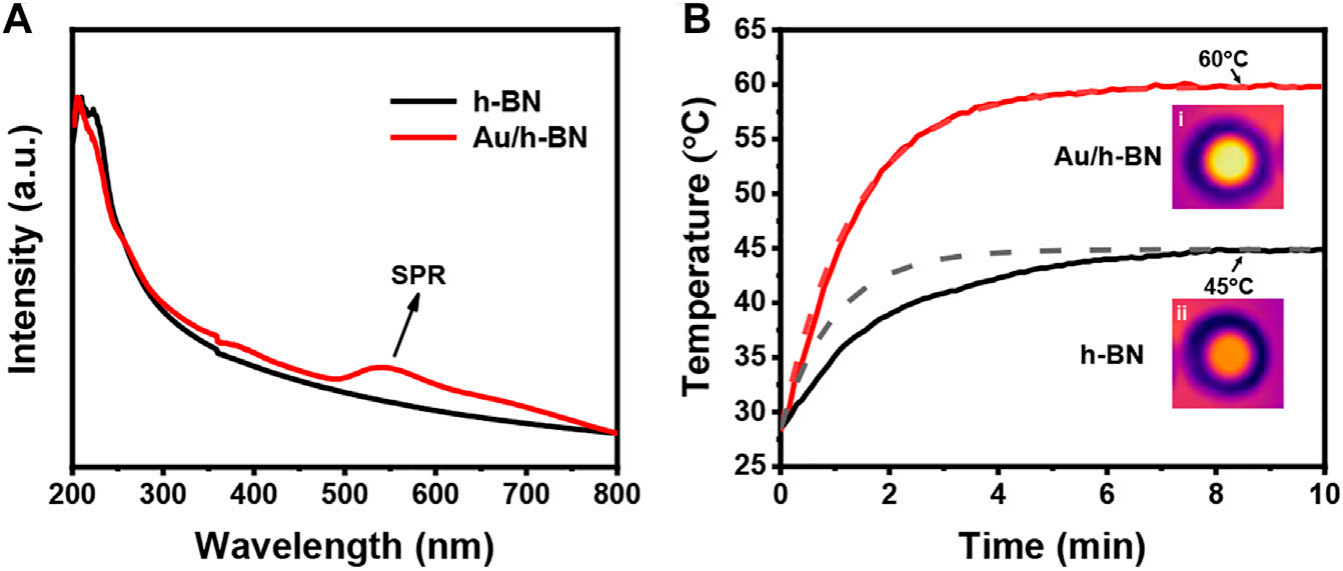
**(A)** UV-vis spectra for Au/h-BN composite and bare h-BN. **(B)** Temperature-time curves for Au/h-BN suspension and h-BN suspension. Solid curves and dash lines correspond to the experimental data and theoretical calculations, respectively. The insets are experimental IR thermal images from top views.

UV-vis spectroscopy was performed to measure the LSPR. As shown in Figure 3A, the LSPR band is centered at 545 nm for Au/h-BN composite, while bare h-BN shows no LSPR features in the visible region. The photothermal property of the samples is the key in this study, which was disclosed by an infrared camera while illuminating the sample solution with a Xe lamp. Figure 3B depicts the temperature-time curves for h-BN and Au/h-BN suspension, and the insets are corresponding IR thermal images after equilibrium (from top views). Under the light irradiation, the temperature of h-BN suspension can reach 45°C, due to light excited and coupled phonons in the lattices (Lopez et al., 2018). Notably, a higher temperature can be obtained on the Au/h-BN composite, an increase of 15°C–60°C due to the plasmonic heating from Au. This temperature is high enough to initiate several reactions. The experimental data was fitted with a well-known theoretical function of temperature rise in photothermal effects (Duong et al., 2018; Phan et al., 2018; Phan et al., 2019): *T*(*t*) = *T*_0_+*A/B* (1-exp(-*Bt*)). The *B* was calculated by plotting ln (*T*-*T*_0_)/(*T*_max_-*T*_0_) vs. time and the A was determined by T_max_ = T_0_+A/B. For h-BN, A_1_ = 16.21, B_1_ = 0.98, it can be found that the deviation between the theoretical curve and experimental values is small. For Au/h-BN, A_2_ = 23.60. B_2_ = 0.75, the fitted curve shows a good agreement with the experimental result.

### Photothermal Catalytic Properties of Au/h-BN

**FIGURE 4:**
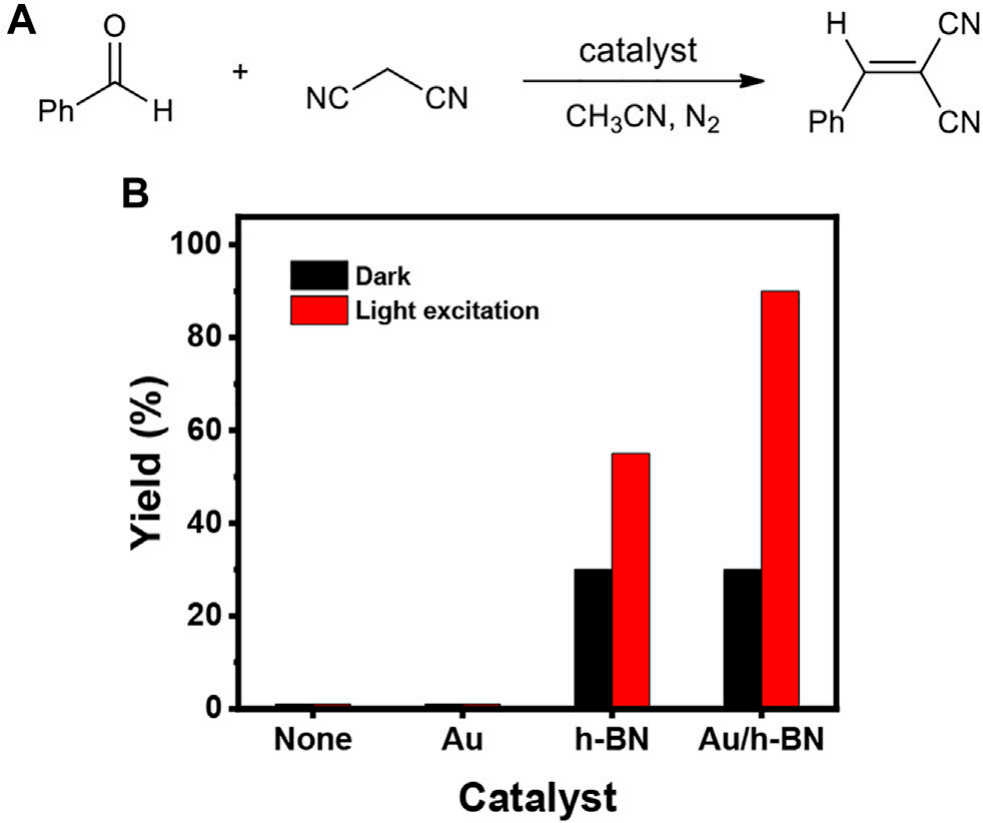
**(A)** Scheme for the reaction of benzaldehyde and malononitrile leading to α-cyanoacrylonitriles. **(B)** Yield percentages using different catalysts; reaction condition: benzaldehyde (0.1 mmol), malononitrile (0.13 mmol), different catalyst (2 mg), CH_3_CN (1 ml), irradiated under a Xe lamp or in the dark for 18 h, in a N_2_ atmosphere.

After ensuring the photothermal effect, the Au/h-BN catalyst was tested for cyanation reactions to establish the catalytic activity. The investigation was carried out by using benzaldehyde as a substrate (0.1 mmol, 10.6 mg, 1 equiv) and malononitrile as a cyanation reagent (0.13 mmol, 8.6 mg, 1.3 equiv) with or without different catalysts (2 mg, 18.8 wt%) in 1 ml CH_3_CN under a N_2_ atmosphere in the dark or under light excitation (Figure 4). When the reaction was performed without catalyst, only trace product was detected after 18 h either in dark or under light irradiation, implying a catalyst for this reaction is required. A similar yield was observed when Au nanoparticles were used as a catalyst, indicating Au (and hot electrons) do not catalyze the reaction under the employed conditions. For the h-BN and Au/h-BN catalysts, the desired product was obtained in the dark in 31 and 30% yields, respectively. This catalytic activity results from the active sites at the hanging bond of the defective h-BN, as discussed above. Under light irradiation, only a limited increase in the product yield is observed for the bare h-BN (from 31 to 55%). In contrast, a remarkable increase is achieved for the Au/h-BN composite (from 30 to 90%), attributed to the plasmonic heating effect from Au, in line with the photothermal results (Figure 3). It is also confirmed that both catalytic h-BN and the light-induced heat are crucial for the high yield of the reaction.

### Proposed Reaction Mechanism

**FIGURE 5:**
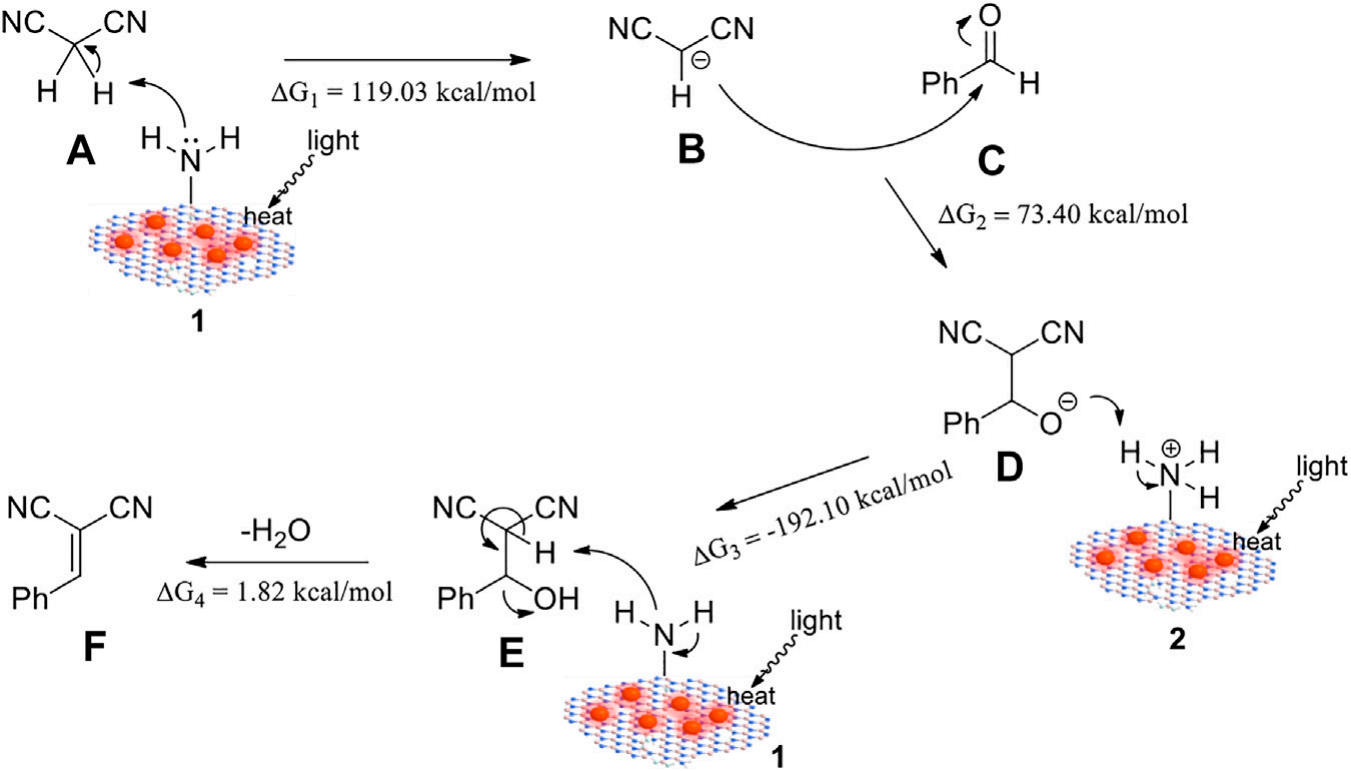
Proposed reaction mechanism for the photothermal reaction using Au/h-BN catalysts.

On the basis of the above results and previous literature (Motokura et al., 2008; Zhang et al., 2016; Lei et al., 2018; Cao et al., 2019; Zhang et al., 2020), a plausible mechanism based on a weak base-catalyzed cycle for this reaction is proposed in Figure 5, applying the -NH_2_ groups as catalytic sites and light-induced heat as the driving force. Firstly, an -NH_2_ group of the catalyst abstracted a proton from malononitrile **A** to generate a carbanion **B**. Then, **B** nucleophilically attacked benzaldehyde **C** to form an intermediate **D**, which further reacted with the protonated catalyst, leading to an intermediate **E**. Finally, **E** eliminated a molecule of H_2_O with the help of catalyst to afford the final product **F**. DFT calculations were performed to illuminate the free energy change in the reaction process. As shown in Figure 5 and Supplementary Figure S6, the free energy change values △*G*_1_ = 119.03 kcal/mol, △*G*_2_ =−73.40 kcal/mol, and △*G*_4_ = 1.82 kcal/mol implied the endothermic steps. Also, the total free energy change △*G*_total_ = 2.15 kcal/mol suggested the total reaction of benzaldehyde and malononitrile leading to α-benzylidenemalononitrile and water was endothermic. The plasmonic heating can provide energy for these processes and thus promote the reaction.

### Generality of the Photothermal Reaction

The generality of the present photothermal protocol was examined. As depicted in Table 1, for all of the substituted aldehydes investigated, desired products were obtained in good yields (Entries 1–8). All of the α-cyanoacrylonitriles products were fully confirmed with ^1^H and ^13^C NMR spectra (see *Conclusion* in Supplementary Material for detail). Furthermore, it is found that the substrates with an electron-donating group such as -CH_3_ or -OCH_3_ (Table 1, Entries 2 and 3) can give higher yields than those with an electron-withdrawing group like -Cl (Table 1, Entries 4 and 5). Notably, the *ortho*-position (2-Cl)-substituted substrates do not hinder the reaction to form the final products in middle yields (Table 1, Entry 5). Moreover, furfural and substituted-furfurals are also compatible in this reaction as well, leading to the corresponding products in high yields (Table 1, Entry 6–8).

**TABLE 1:** Scope of substrates for the photothermal reaction.^a^

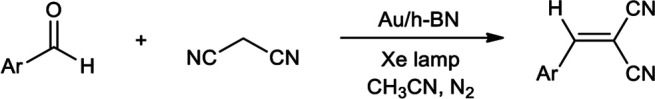			

**Entry**	**Substrate**	**Product**	**Yield (%)** ^b^
1	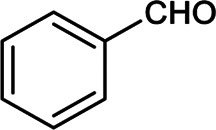	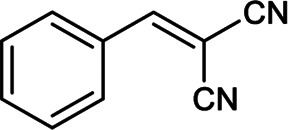	90
2	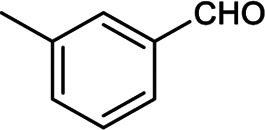	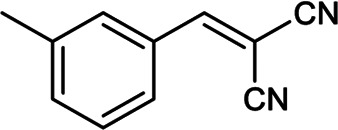	91
3	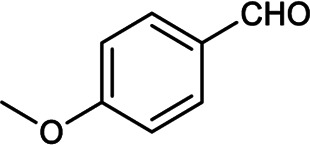	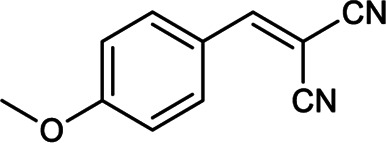	95
4	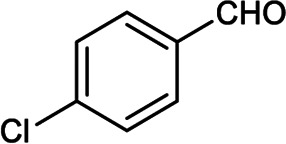	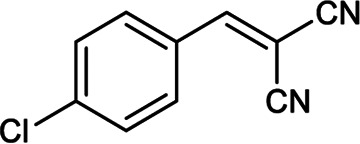	43
5	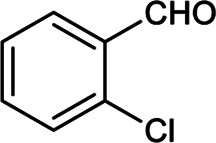	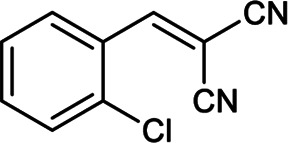	36
6	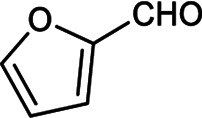	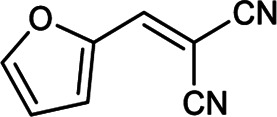	93
7	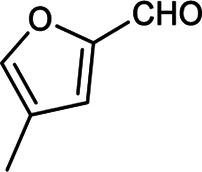	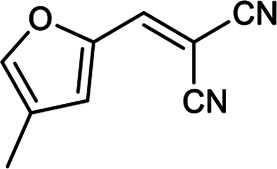	93
8	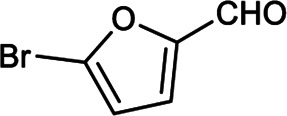	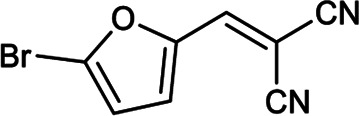	85

a Reaction condition: aldehydes (0.1 mmol), malononitrile (0.13 mmol), Au/h-BN catalyst (2 mg), CH_3_CN (1 ml), irradiated under a Xe lamp for 18 h, in a N_2_ atmosphere.

b Isolated yield.

## Conclusion

In summary, we have demonstrated the Au/h-BN nanocomposite as a promising photothermal catalyst for the synthesis of α-cyanoacrylonitriles. The Au/h-BN composite coupling the plasmonic heating of Au with the catalytic sites of defect-rich h-BN exhibits great catalytic activity in synthesizing α-cyanoacrylonitriles with a broad scope of substrates under mild conditions. As compared to the dark condition, the yield of the cyanation reactions can be greatly enhanced on Au/h-BN composite under light irradiation, which manifests the importance of plasmonic heating. Further development of new photothermal catalysis is under investigation in our lab.

## Data Availability

The original contributions presented in the study are included in the article/Supplementary Material, further inquiries can be directed to the corresponding authors.
