# Supplementary material for: Plasmonic Heating-Promoted Photothermal Synthesis of α-Cyanoacrylonitriles Over Au/h-BN Catalysts
Source: Front Chem. 2021 Sep 8;9:732162. doi: 10.3389/fchem.2021.732162 (PMC8455885; doi:10.3389/fchem.2021.732162)
Supplement: Supplementary file 1 [file DataSheet1.PDF]

Supplementary Material

Plasmonic Heating-Promoted Photothermal Synthesis of α-Cyanoacrylonitriles Over Au/h-BN Catalysts

Ce Liang[1], Yuanyuan Zhang[1], Bin Zhang[1], Xin-Miao Liu[1], Guo-Lin Gao[1], Jingyan Cao[2]* and Ping Xu[1]*

[1] MIIT Key Laboratory of Critical Materials Technology for New Energy Conversion and Storage, School of Chemistry and Chemical Engineering, Harbin Institute of Technology, Harbin 150001, P. R. of China

[2] Department of Medical Oncology, Harbin Medical University Cancer Hospital, Harbin 150081, P. R. of China

*E-mail: pxu@hit.edu.cn (P.X.); caojngyan@126.com (J.C.)

Contents

1. Supporting characterization for Au nanoparticles, defect-rich h-BN, and Au/h-BN composite . 2

2. Equations and methods for calculations of yields 4

3. Optimized molecular structures 5

4. Characterization data of α-cyanoacrylonitriles products 6

1. Supporting characterization for gold nanoparticles, defect-rich h-BN, and Au/h-BN composite

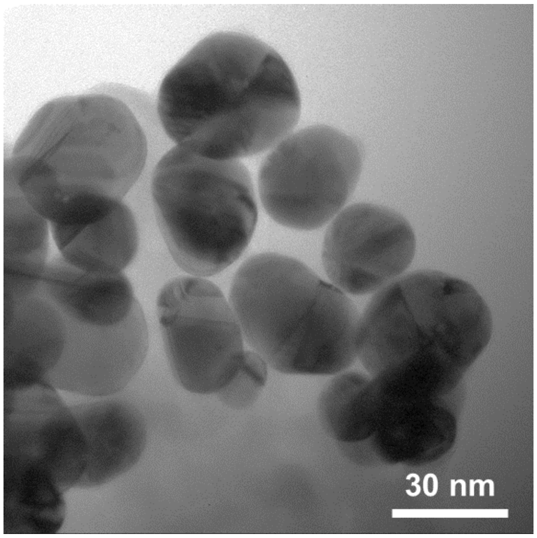

FIGURE S1 | TEM image for the prepared gold nanoparticles. The morphology of Au nanoparticles was nanospheres with diameters of ~30 nm.

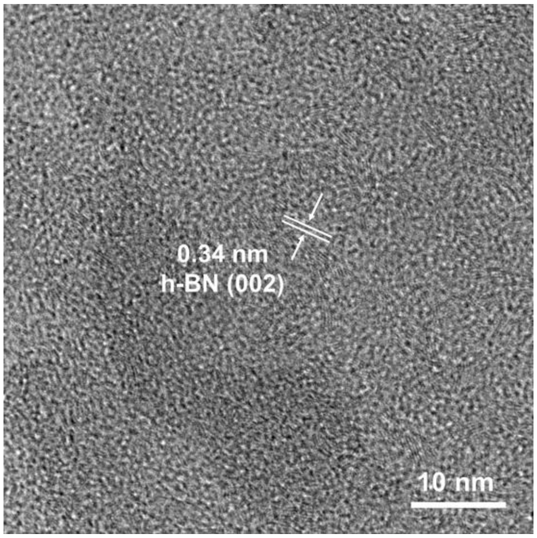

FIGURE S2 High-resolution TEM image for the defect-rich h-BN. The irregular lattice fringes revealed a disorder of crystallization, corresponding to the XRD results. The interlayer distance was about 0.34 nm, indexed to the interplane (002) spacing of h-BN.[1]

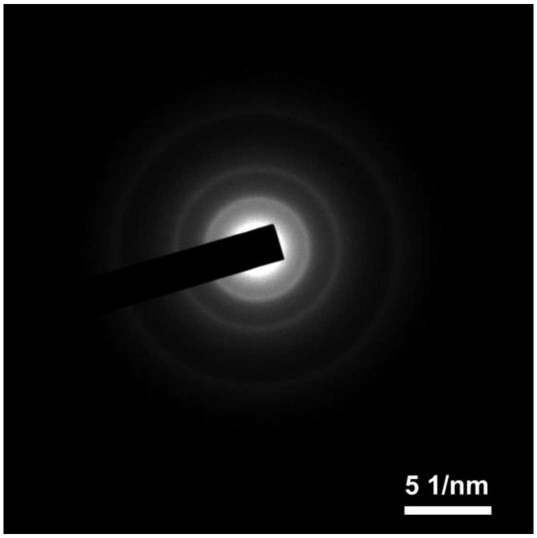

FIGURE S3 Selected-area electron diffraction (SAED) pattern for defective h-BN sheets. The SAED pattern showed three reflections corresponding to (002), (100), and (110) planes of h-BN (from the inside out). The diffused diffraction rings suggested the defective and polycrystalline nature of the sheets, in agreement with high-resolution TEM observations.[1]

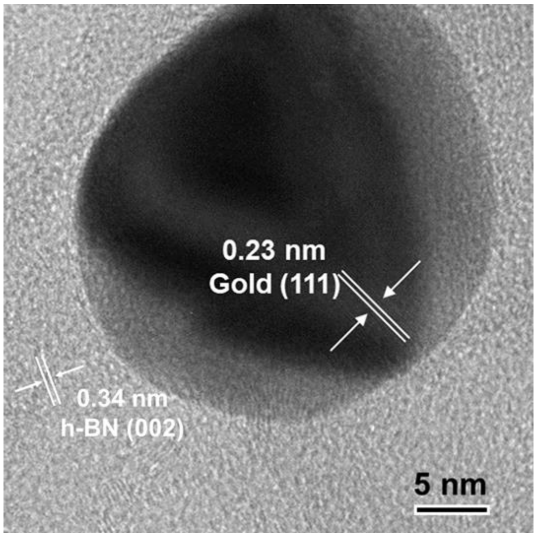

FIGURE S4 High-resolution TEM image for the Au/h-BN composite. Two lattice fringes of 0.23 nm and 0.34 nm were revealed in this image, which matched well with the (111) facet of the Au and (002) facet of h-BN, respectively. This was evidence for the formation of the Au/BN composite.[1,2]

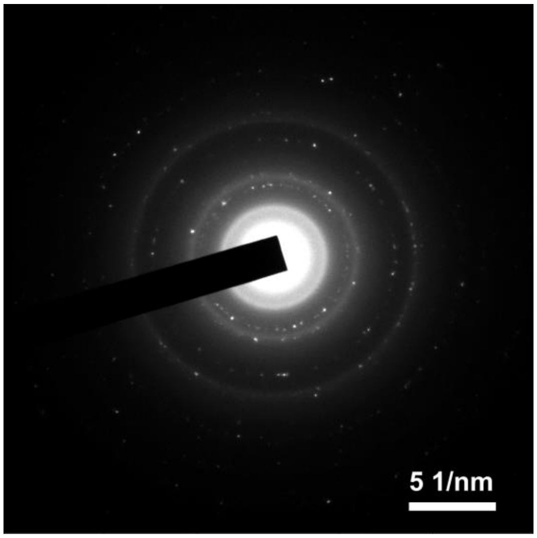

FIGURE S5 SAED pattern for Au/h-BN composite. The diffraction rings caused by both gold and h-BN could be observed simultaneously, consistent with XRD results. This could confirm Au/h-BN composite as well.[1,2]

2. Equations and methods for calculations of yields

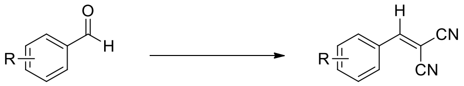

	substrates	products
	aldehydes	α-cyanoacrylonitriles
equivalent	1	1
molar	0.1 mmol	0.1 mmol
molecular weight		M mg/mmol
weight^1 (theoretical value)		0.1 mmol * M mg/mmol
= molar*molecular weight		= 0.1M mg

The weight of the isolated product from the experimental result was denoted as "weight^2", it can be measured by using an analytical balance.

Thus, the Yield = weight^2 (experimental result) / weight^1 (theoretical value) *100%= weight^2/(0.1M mg) *100%

3. Optimized molecular structures

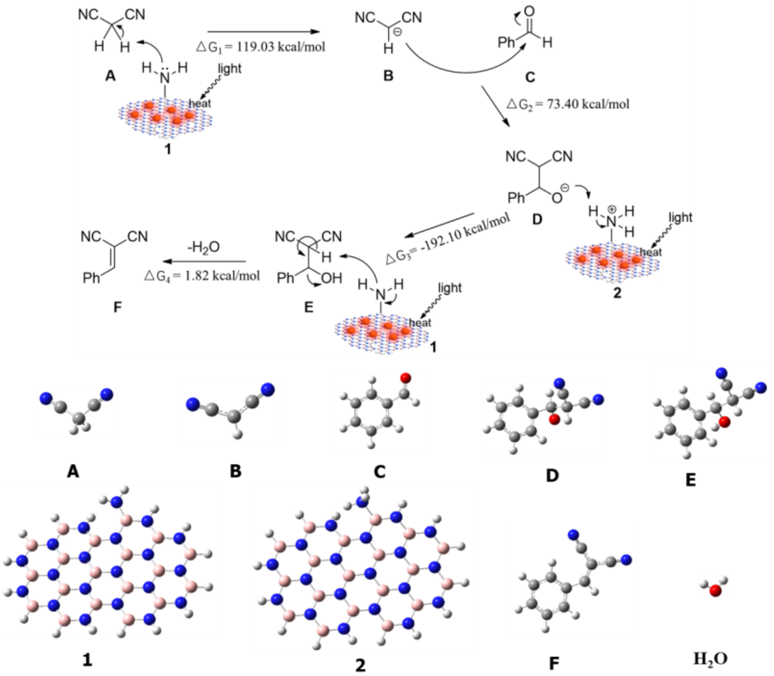

$\triangle G_1=G_B+G_2-G_A-G_1=$ 119.03 kcal/mol

$\triangle G_2=G_D-G_B-G_C=$ 73.40 kcal/mol

$\triangle G_3=G_E+G_1-G_D-G_2=$ -192.10 kcal/mol

$\triangle G_4=G_F+G_{H_2O}-G_E=$ 1.82 kcal/mol

$\triangle G_{total}= \triangle G_1+\triangle G_2+\triangle G_3+\triangle G_4$

$= G_F+G_{H_2O}-G_A-G_C=2.15$ kcal/mol

No.	G (kcal/mol)
A	-141149.12
B	-140808.45
C	-216787.54
D	-357522.59
E	-357936.33
F	-309993.77
H_2O	-47940.74
1	-990975.48
2	-991197.12

FIGURE S6 Optimized molecular structures in the reaction process and corresponding free energy. B: pink; N: blue; C: grey; O: red; H: white. To facilitate the calculations of free energy change in the reactions, Au nanoparticles were omitted during the calculations because the experimental results showed that the Au nanoparticles only act as nanoheaters rather than catalytic sites.

4. Characterization data of α-cyanoacrylonitriles products

2-benzylidenemalononitrile. ^{1}H NMR (400 MHz, $CDCl_3$) δ 7.91 (d, J = 7.8 Hz, 2H), 7.79 (s, 1H), 7.64 (t, J = 7.4 Hz, 1H), 7.55 (t, J = 7.6 Hz, 2H). ^{13}C NMR (101 MHz, $CDCl_3$) δ 159.90, 134.57, 130.87, 130.66(2C), 129.57(2C), 113.65, 112.49, 82.79.

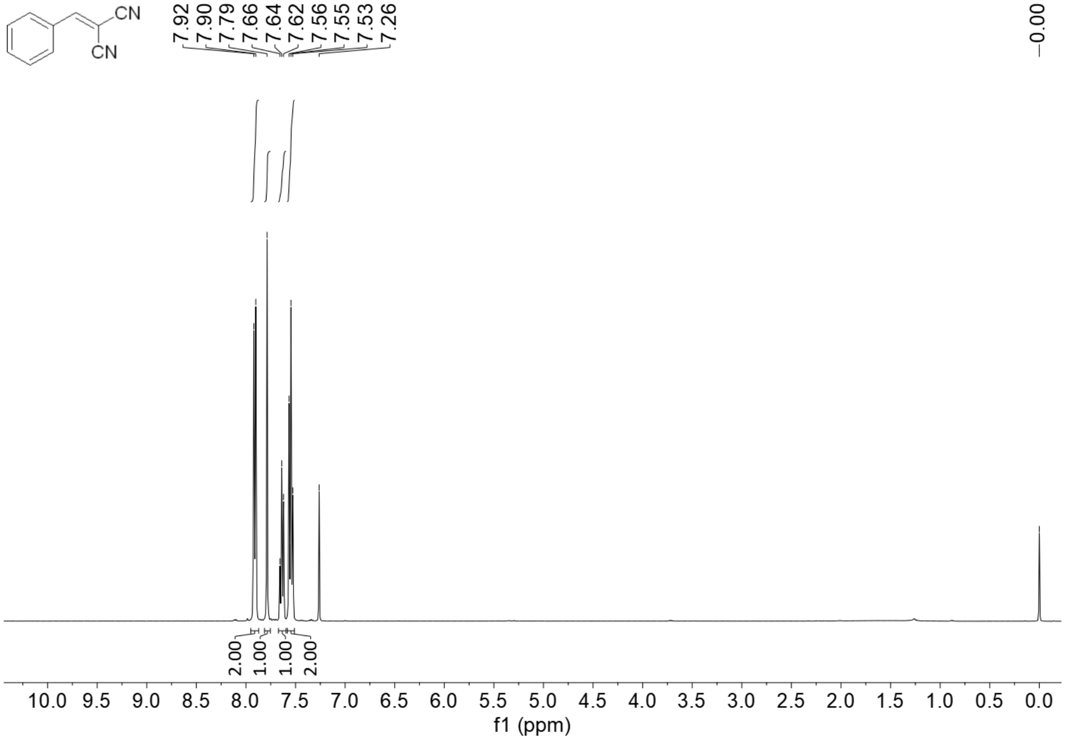

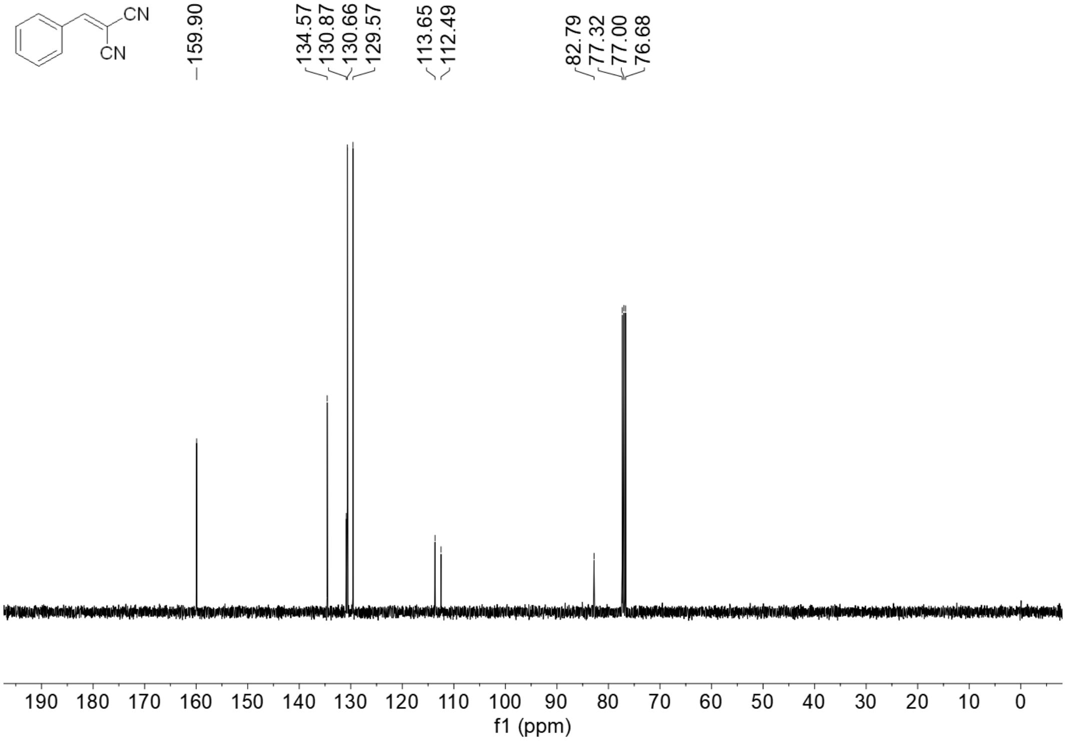

2-(3-methylbenzylidene)malononitrile. ^{1}H NMR (600 MHz, $CDCl_3$) δ 7.74-7.73 (m, 2H), 7.69 (s, 1H), 7.45-7.41 (m, 2H), 2.43 (s, 3H). ^{13}C NMR (151 MHz, $CDCl_3$) δ 160.13, 139.58, 135.53, 131.23, 130.88, 129.45, 127.86, 113.79, 112.59, 82.33, 21.22.

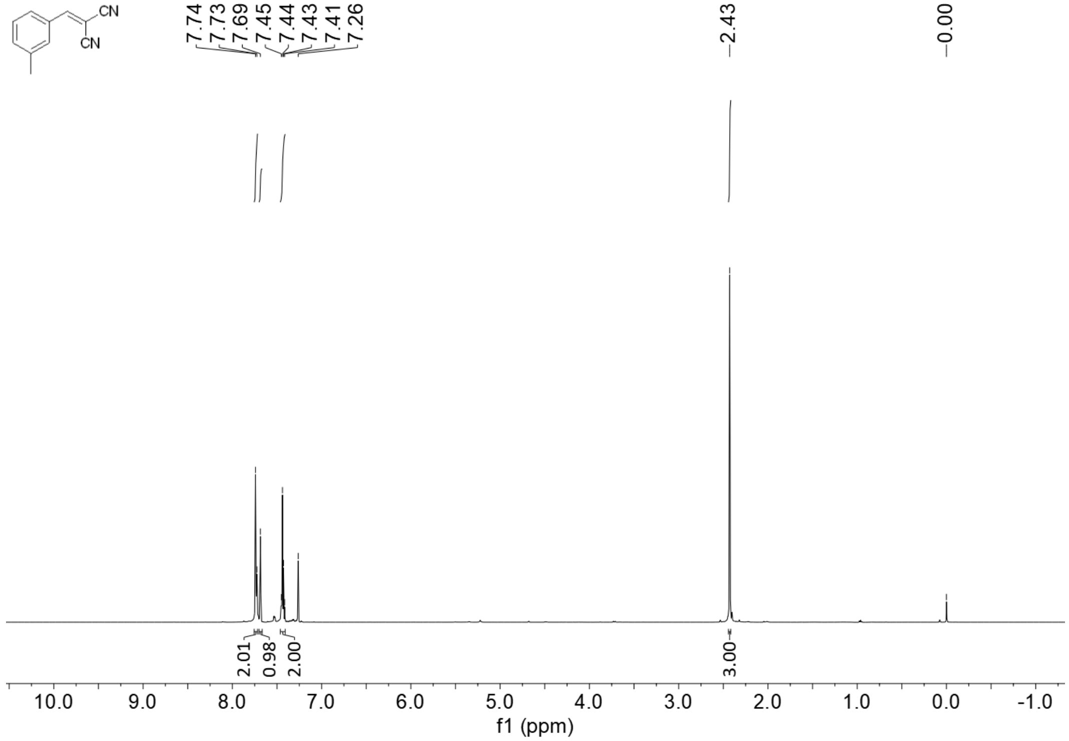

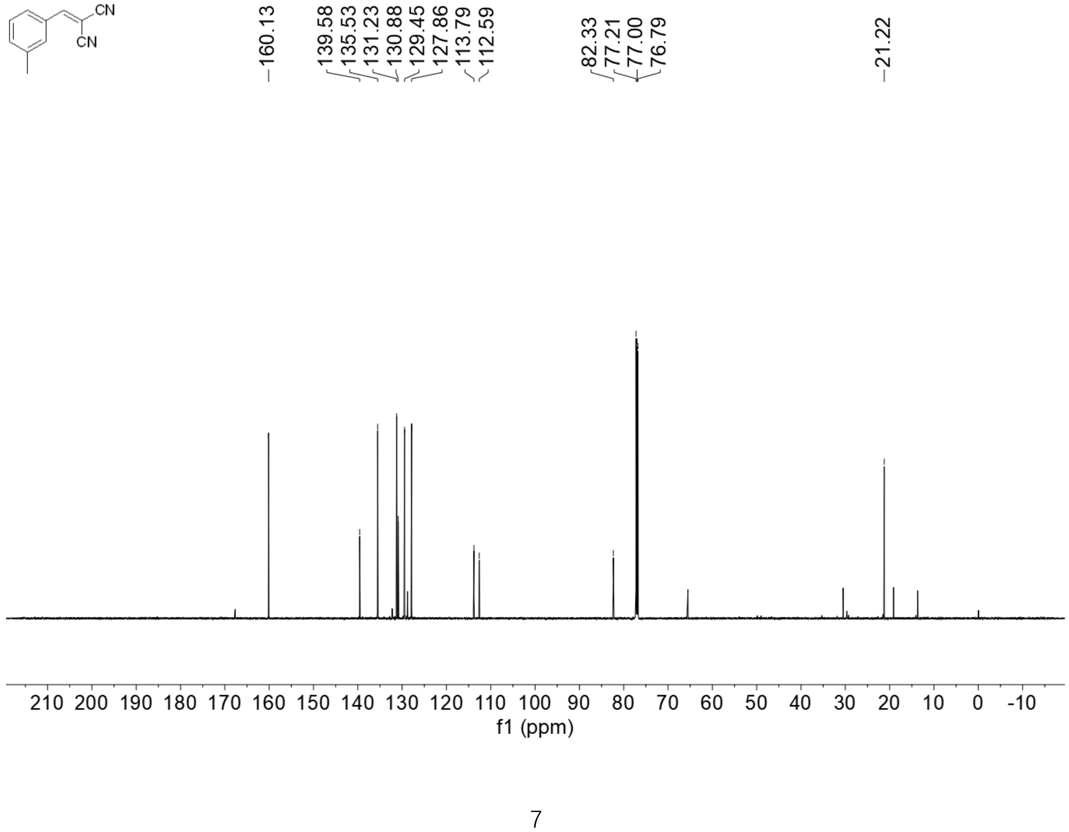

2-(4-methoxybenzylidene)malononitrile. ^{1}H NMR (400 MHz, $CDCl_3$) δ 7.91 (d, *J* = 8.6 Hz, 2H), 7.66 (s, 1H), 7.02 (d, *J* = 8.6 Hz, 2H), 3.92 (s, 3H). ^{13}C NMR (101 MHz, $CDCl_3$) δ 164.77, 158.82, 133.39, 123.95, 115.07, 114.38, 113.30, 78.41, 55.74.

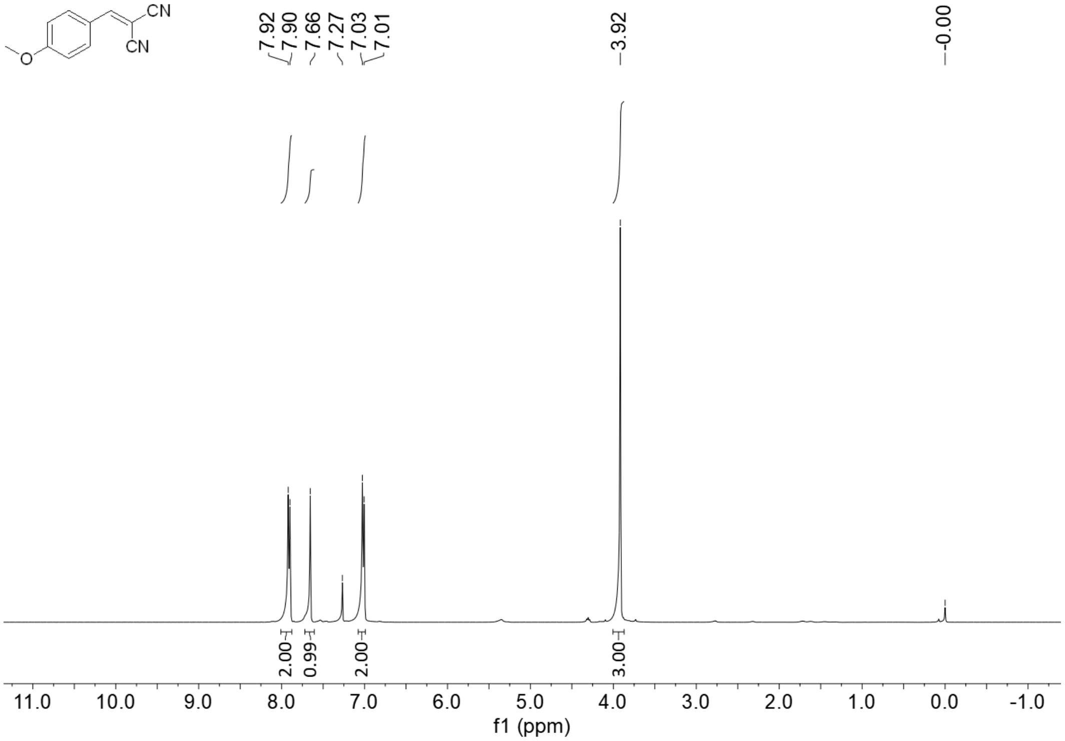

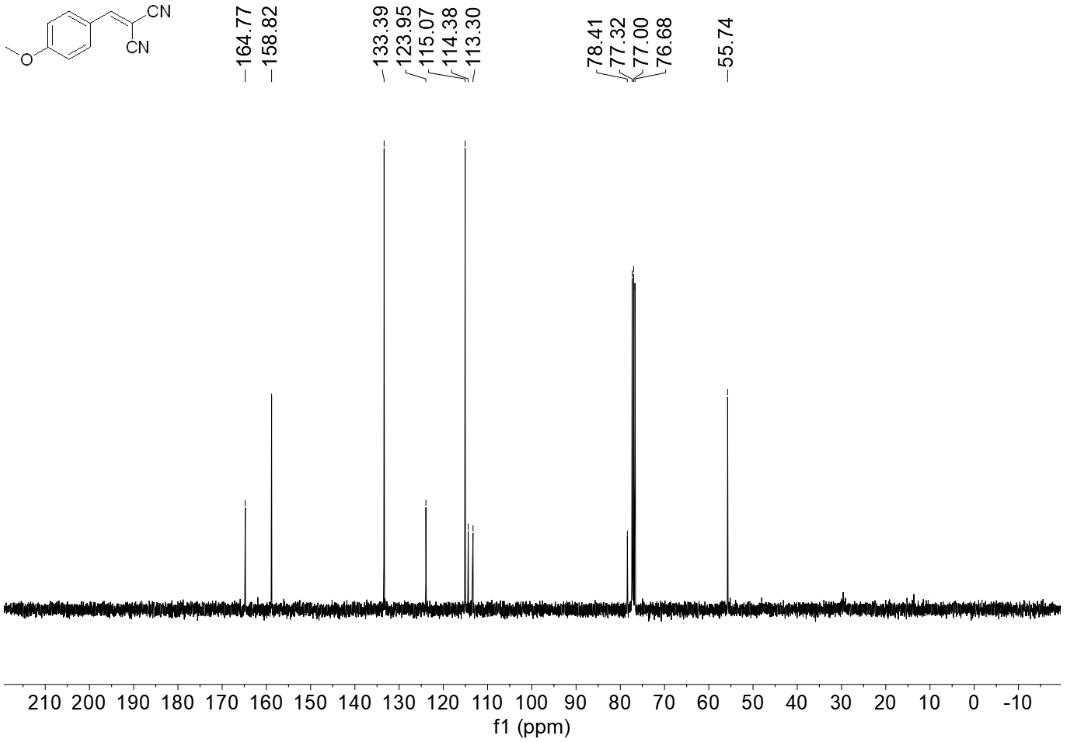

2-(4-chlorobenzylidene)malononitrile. ^{1}H NMR (400 MHz, $CDCl_3$) δ 7.85 (d, J = 8.4 Hz, 2H), 7.74 (s, 1H), 7.52 (d, J = 8.5 Hz, 2H). ^{13}C NMR (101 MHz, $CDCl_3$) δ 158.31, 141.11, 131.80, 130.02, 129.23, 113.42, 112.30, 83.25.

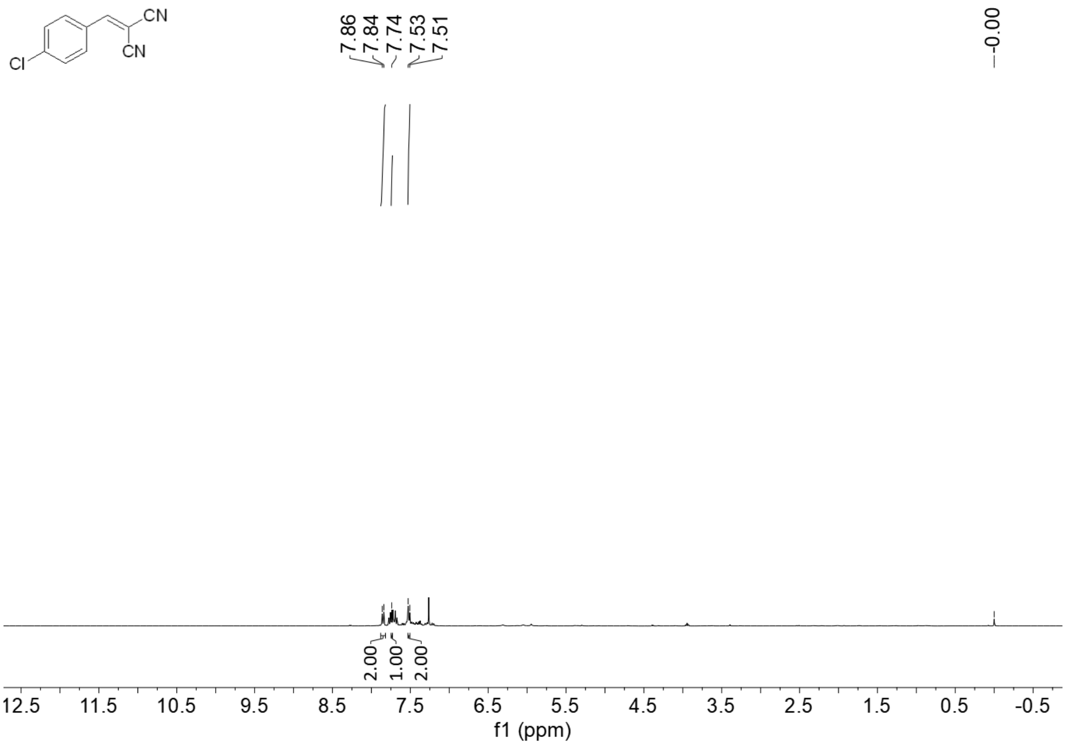

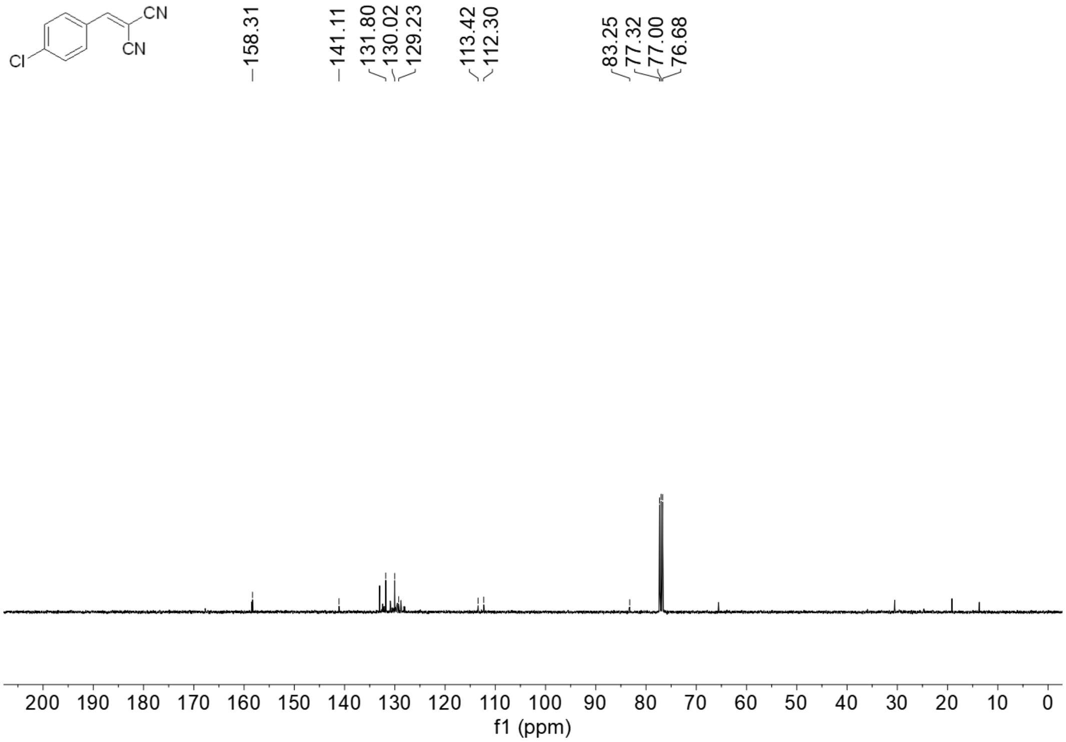

2-(2-chlorobenzylidene)malononitrile. ^{1}H NMR (400 MHz, $CDCl_3$) δ 8.27 (s, 1H), 8.18 (d, *J* = 7.9 Hz, 1H), 7.56 (d, *J* = 4.3 Hz, 2H), 7.47-7.43 (m, 1H). ^{13}C NMR (101 MHz, $CDCl_3$) δ 155.98, 136.28, 134.98, 130.65, 129.44, 129.00, 127.73, 113.15, 111.85, 85.74.

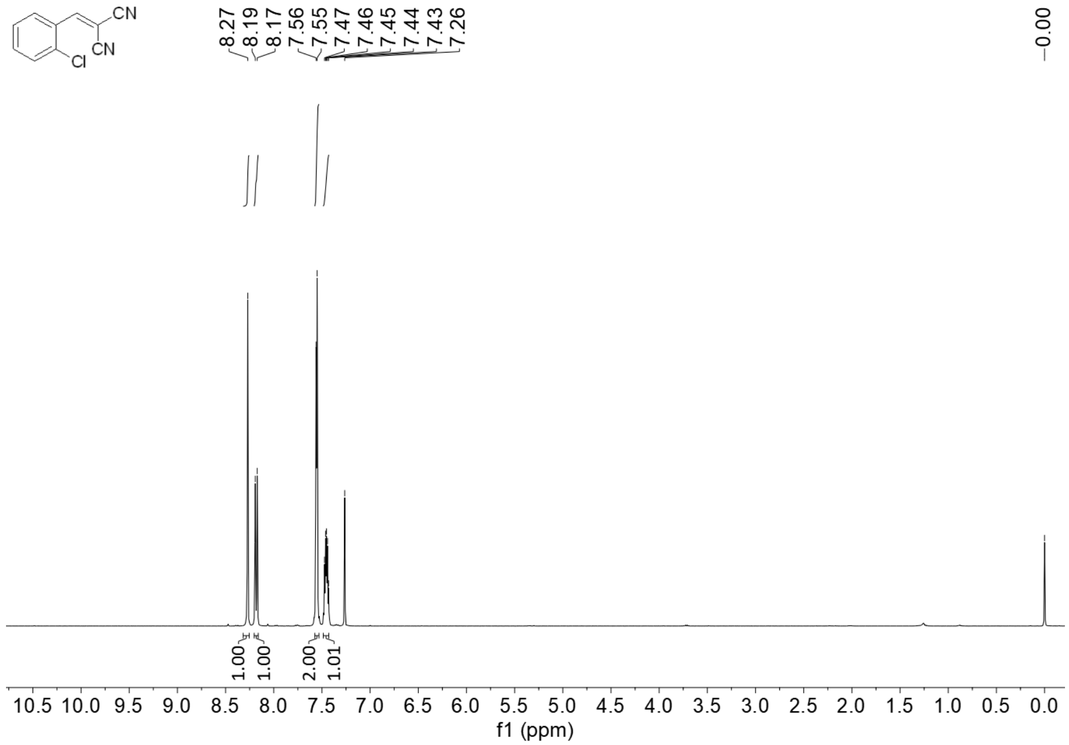

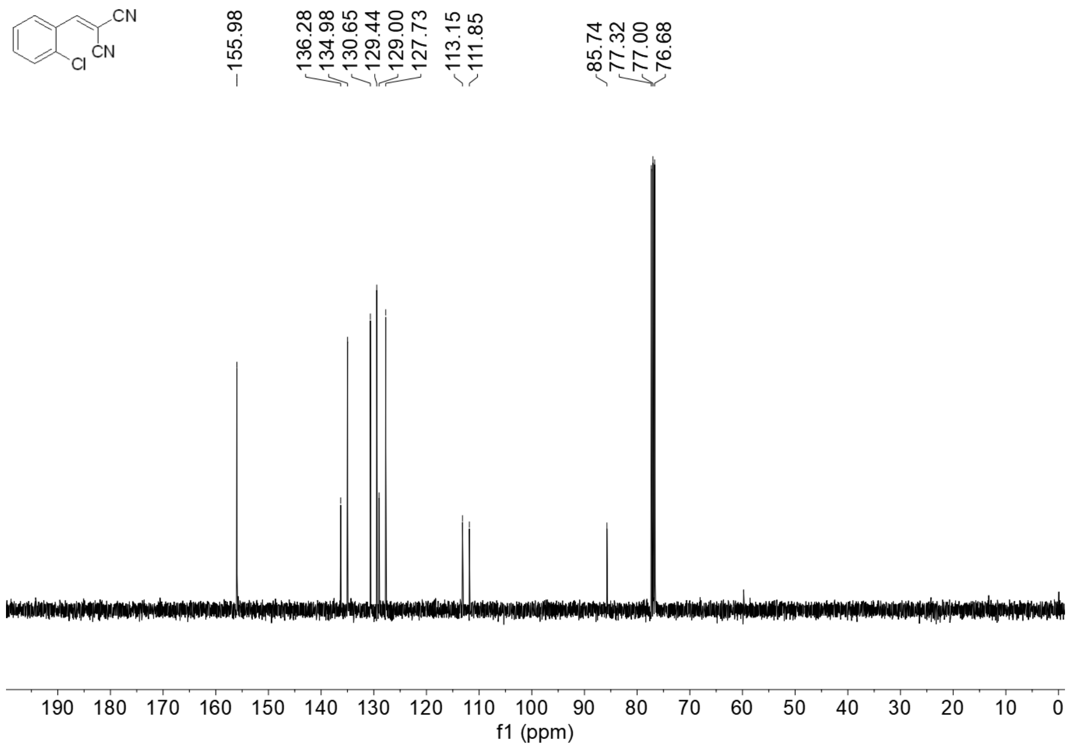

2-(furan-2-ylmethylene)malononitrile. ^{1}H NMR (400 MHz, $CDCl_3$) δ 7.82 (s, 1H), 7.55 (s, 1H), 7.37 (d, J = 2.6 Hz, 1H), 6.74 – 6.73 (m, 1H). ^{13}C NMR (101 MHz, $CDCl_3$) δ 149.50, 147.89, 142.98, 123.58, 114.32, 113.71, 112.50, 77.11.

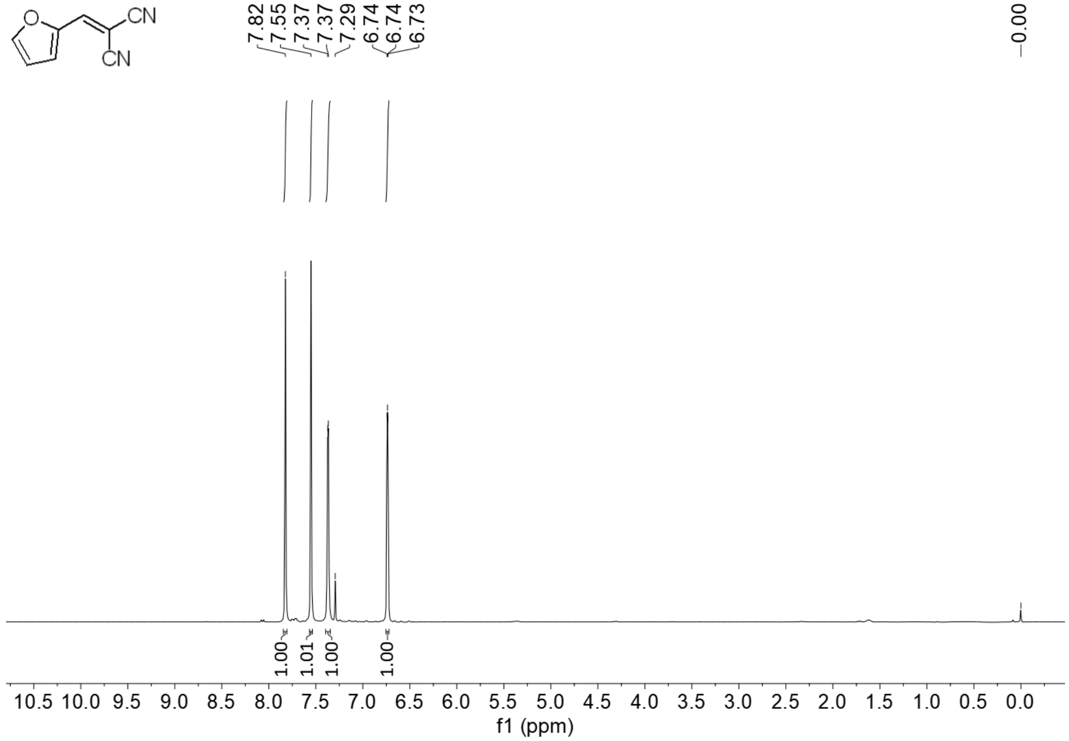

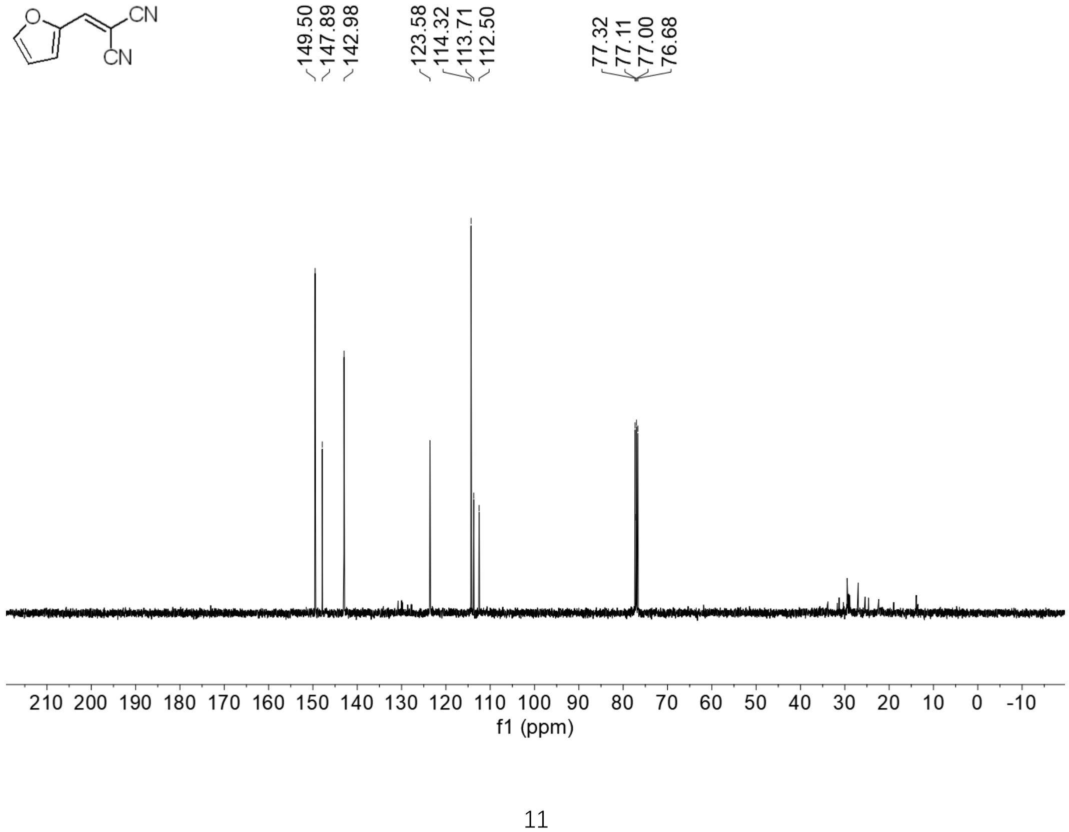

2-((5-methylfuran-2-yl)methylene)malononitrile. ^{1}H NMR (400 MHz, $CDCl_3$) δ 7.40 (s, 1H), 7.28 (d, *J* = 7.6 Hz, 1H), 6.39 (d, *J* = 3.6 Hz, 1H), 2.48 (s, 3H). ^{13}C NMR (101 MHz, $CDCl_3$) δ 161.91, 146.85, 142.10, 125.84, 114.28, 112.96, 111.72, 74.23, 14.20.

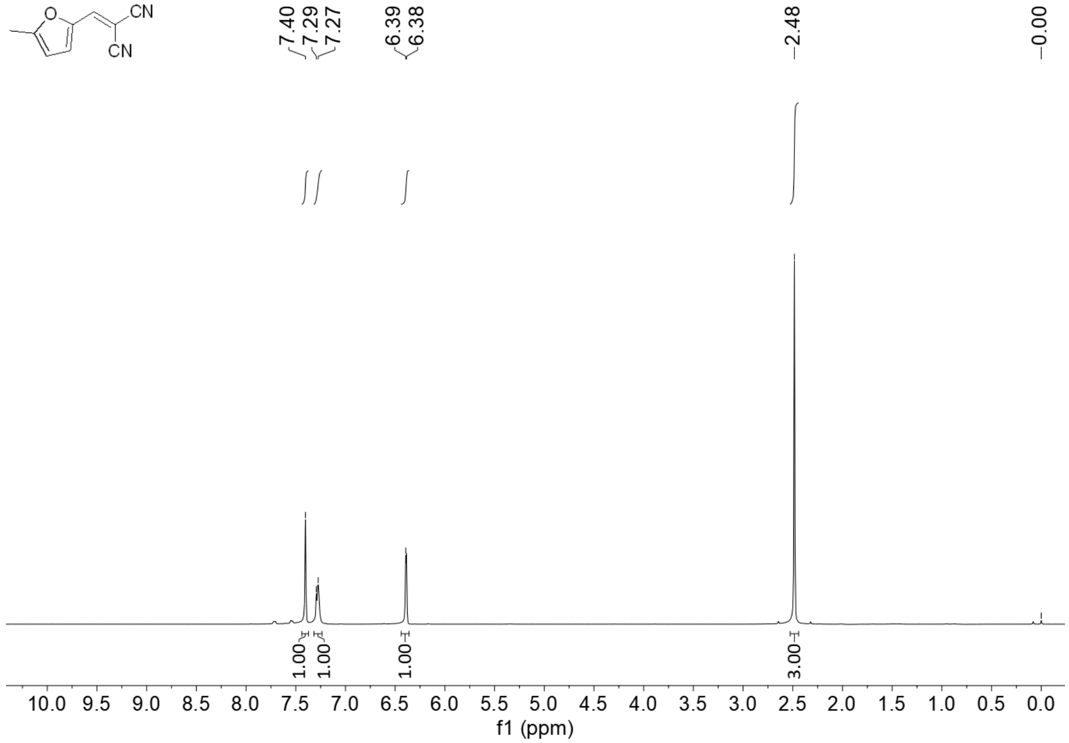

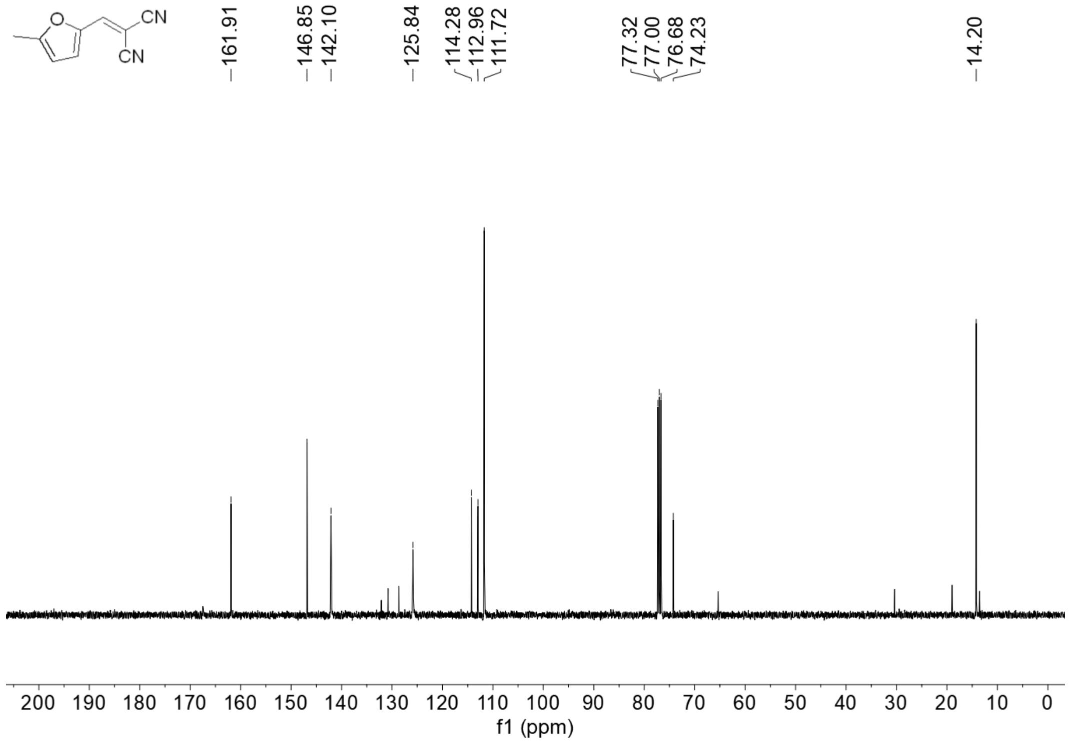

2-((5-bromofuran-2-yl)methylene)malononitrile. ^{1}H NMR (400 MHz, $CDCl_3$) δ 7.44 (s, 1H), 7.35 (d, J = 3.8 Hz, 1H), 6.68 (d, J = 3.8 Hz, 1H). ^{13}C NMR (101 MHz, $CDCl_3$) δ 149.65, 141.49, 132.47, 124.84, 116.63, 113.50, 112.28, 77.61.

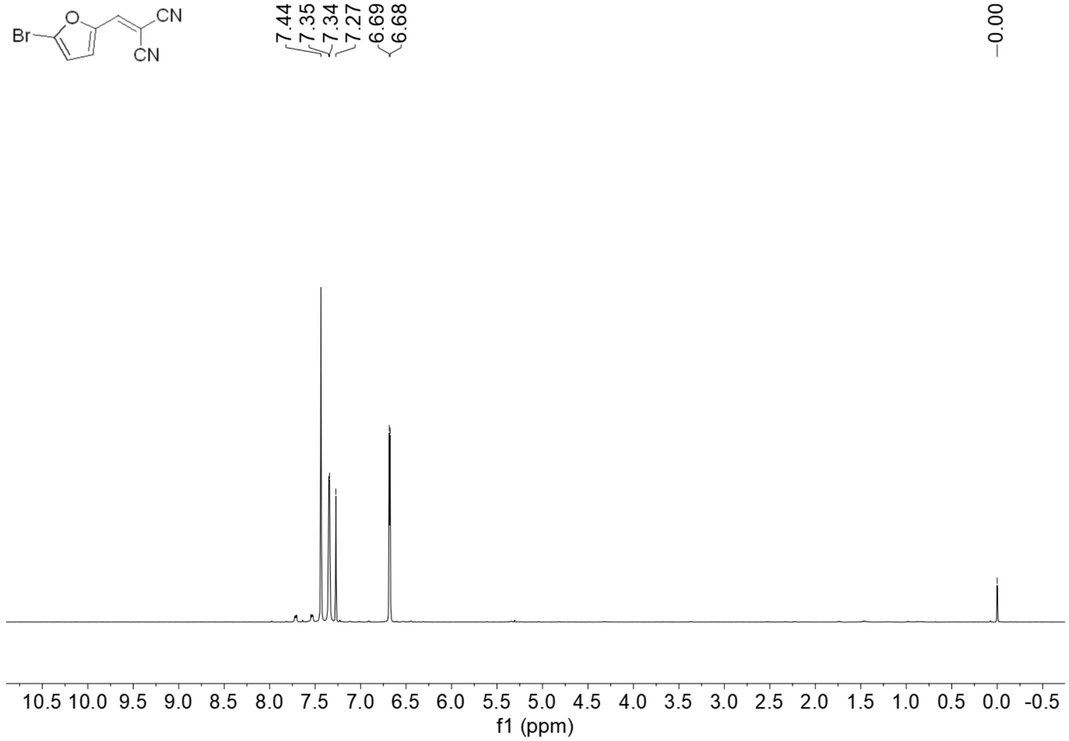

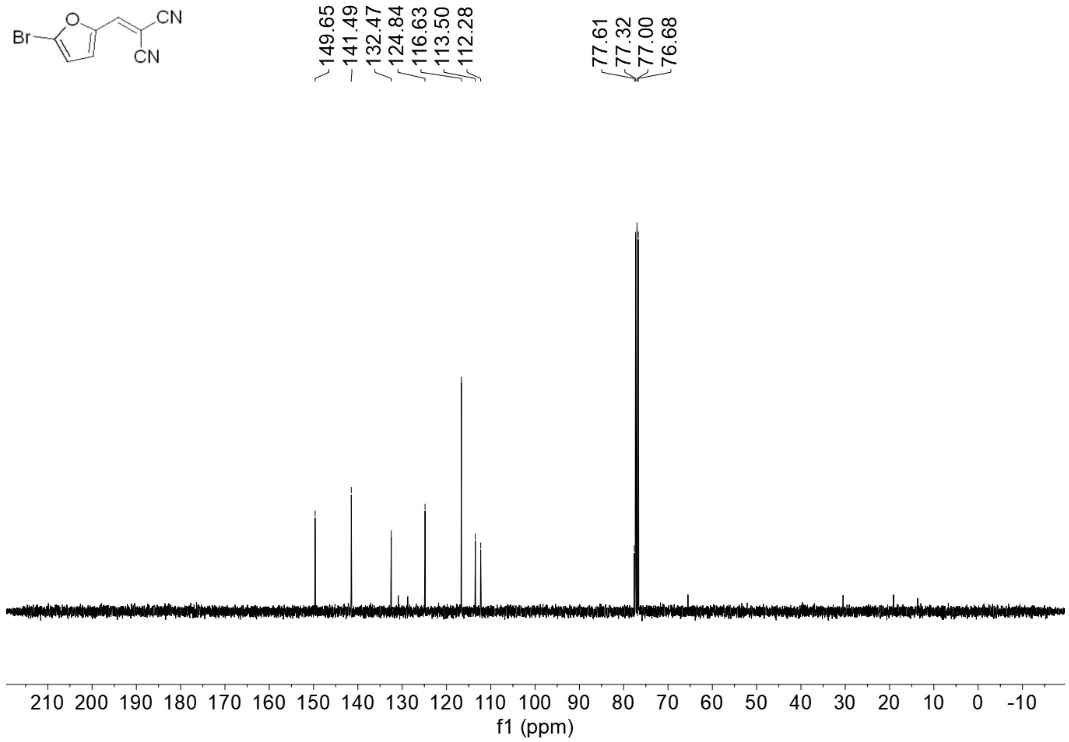

Reference

(1) Hong, S.; Lee, C. S.; Lee, M. H.; Lee, Y.; Ma, K. Y.; Kim, G.; Yoon, S. I.; Ihm, K.; Kim, K. J.; Shin, T. J.; Kim, S. W.; Jeon, E. C.; Jeon, H.; Kim, J. Y.; Lee, H. I.; Lee, Z.; Antidormi, A.; Roche, S.; Chhowalla, M.; Shin, H. J.; Shin, H. S. Ultralow-Dielectric-Constant Amorphous Boron Nitride. *Nature* **2020,** *582*, 511-514.

(2) Depciuch, J.; Stec, M.; Kandler, M.; Baran, J.; Parlinska-Wojtan, M. From Spherical to Bone-Shaped Gold Nanoparticles-Time Factor in the Formation of Au NPs, Their Optical and Photothermal Properties. *Photodiagnosis Photodyn. Ther.* **2020,** *30*, 101670.
